# The Relation of Tuberculosis in Domesticated Animals and Man1Read before the Washington State Medical Society at Spokane, May 9th, 1900.

**Published:** 1900-08

**Authors:** Sofus B. Nelson

**Affiliations:** Professor of Veterinary Science, Washington Agricultural College and School of Science


### THE RELATION OF TUBERCULOSIS IN DOMESTICATED ANIMALS AND MAN.[1]

BY SOFUS B. NELSON, D.V.M.,
PROFESSOR OF VETERINARY SCIENCE, WASHINGTON AGRICULTURAL COLLEGE AND SCHOOL OF SCIENCE.

SINCE Koch, in 1882, demonstrated the bacillus tuberculosis to be the specific cause of tuberculosis, this disease has been a source of constant study. Without doubt it is one of the most important diseases with which man has to contend. Judging from the number of scientists that are laboring to unravel some one of the many phases of tuberculosis, of interest to the welfare of mankind, to which Koch's discovery was the first key, this importance seems to be indeed realized. Nearly all animals are susceptible to this disease. Among our domestic animals the cow, horse, sheep, pig, dog, cat, and chicken succumb to it. It has been observed in wild animals, such as the lion, tiger, bear, giraffe, monkey, etc., that have been placed

[1] Read before the Washington State Medical Society at Spokane, May 9th, 1900.

and kept in confinement in zoological gardens. That rodents, especially in densely populated districts, are seriously affected has been demonstrated.

That certain wild animals, when kept in close confinement, contract and succumb to tuberculosis is not very important to us except as a fact; but that our domestic animals—our dumb friends, our source of various foods and material for our comforts and luxuries—are subject to this disease is of great importance, and deserves careful and thorough consideration. Of all the domestic animals the cow is the most susceptible to tuberculosis. In the others it is not found nearly so frequently. It is quite a rare disease in swine, as the following statistical statement shows: At Augsburg, out of 30,922 swine slaughtered, only 3 were affected.

Considering the domestic animals from a standpoint of economic importance, because of their individual utility to man, the cow stands first in the list, as she has all the points possessed by the other animals, and some that they do not. Here, then, is the cow, the most serviceable animal to man, and at the same time the most susceptible to a certain contagious disease.

The associating together of these two facts would not deserve a moment's serious thought if the contagious disease, tuberculosis, which affects the cow was not transmissible to man, and *vice versa*.

Before discussing this phase of the question it may be advisable to see whether or not the disease is the same in the different lower animals. Investigators are unanimous that tuberculosis in the lower mammals is the same, *i. e.*, that the bacillus tuberculosis is the bacterium that causes the disease. There has, however, been a great deal of discussion over the question whether the bacillus that causes avian and mammalian tuberculosis is identical. Rivolta, Maffuci, Strauss, and others have held that they were not identical, either in morphology or pathogenesis, since the bacillus of avian tuberculosis will grow at 43° C., while that of mammalian will not. Also that it is very rarely that tuberculosis of mammals is transmitted to chickens. Finally, that certain mammals, as the dog and guinea-pig are not susceptible to the avian form. These arguments have been met by Nocard and other, in that the tuberculosis produced by the two bacilli were identical in clinical and post-mortem appearances. Numerous experiments have proven that tuberculosis is transmissible from one of the lower animals to the other. It remains now to be proven whether or not, tuberculosis is transmissible from man to the lower animals and back again. To prove or disprove the transmissibility from man to the lower animals is comparatively easy, as material wherewith to carry out these experiments is easily obtained. But to prove that tuberculosis may or may not be transmitted to man, is a more difficult matter, as in this case it is not possible to carry out direct experiments.

Chauveau, Kruse, and other have shown experimentally that tuberculosis may be transmitted from man to the lower animals, especially to the ox. There are many cases of accidental infections recorded. For instance, that sometimes tuberculosis is caused in chickens, either by inoculations with human cultures, or by the chickens obtaining expectorations from human tuberculous patients. Veterinarian T. A. Bray, of Kansas City, reports an observation of young chickens becoming tuberculous under the latter condition. I have already stated that certain authorities claim that there are two causes of tuberculosis, the two bacilli being individually different. However, Nocard has, by growing cultures of human tubercle bacillus *in vivo*, succeeded in intensely modifying the characters of the same, so that the dry, scaly cultures that are pathogenic to the dog and guinea-pig, have become soft, unctuous and easily handled on media, and do not affect the guinea-pig. Yet the first culture will not cause tuberculosis in the chicken; this not occurring until the bacillus has passed through the chicken three times. This is very strong evidence that the two bacilli are simply two varieties of the same species. Theobald Smith also records the observation of two varieties of the tubercle bacilli from two mammals, one being from a member of the bear family (*Nasua naria*), the other from a tuberculous bull. They differed especially in the pathogenic power toward rabbits.

Concerning the infection of man from the lower animals, there are a number of authentic cases of local cutaneous inoculations of tuberculosis occurring in veterinarians, demonstrators of anatomy and others. Senn, in his *Principles of Surgery*, makes reference to this fact. It is still more difficult to obtain clear and definite evidence of infection in man from the ingestion of food furnished from tuberculous animals. Here, however, Dr. Law, of Cornell, has collected the statistics of a large number of cases recorded by Lydtin; Demme, of Berne; Howe, of North Hadley, Mass.; Dr. Brown, of Ithaca, N. Y.; Ernst, in his *Infectiousness of Milk*, and others, where patients died from tuberculosis which could hardly be explained in any other manner, considering their environments, than that the disease was caused through the direct use of infected food-material obtained from cattle. In addition to this is the strong argument that wherever tuberculosis is extensive in cattle, the same is true in man. Where, in any part of the world, cattle are absent, and, therefore, tuberculosis, as far as they are concerned, man is also comparatively free from it. Although not positively proven that tuberculosis is transmissible from the lower animals to man, especially through the various foods furnished by the cow, yet the generalized evidence is so great that it is dangerous to doubt it.

The danger of using the products of a tuberculous animal for food varies according to the degree the animal is affected and the organ affected. Take, for instance, an animal that has generalized tuberculosis, neither her milk nor flesh is fit for food; but if an animal has simply a slight lesion of a gland then the meat, when well cooked, may be perfectly safe for consumption. If the udder is affected the milk is more likely to contain the bacillus than when the udder is not affected, in which case the milk is more dangerous to use in its raw form.

All of this leads up to the question of prevalence of the disease in man and animals. Regarding the prevalence of the disease in man I can do no better than to quote the distinguished ex-President of the Iowa State Medical Society, Dr. J. M. Emmert, who says: "Take as an illustration tuberculosis, or the 'Great White Plague,' as it is called, one out of every seven deaths throughout the world is caused by tuberculosis. Iowa contributes nine deaths every day, and three thousand during the year; the United States one hundred and fifty thousand yearly, and the world five millions per year. Contemplate these figures for a minute, and then ask yourselves why the people are not aroused to the dangers that surround them, and demand that our law-makers take some action to protect them."

In our own State there were last year 242 deaths from tuberculosis reported to the office of the Secretary of the State Board of Health, the total number of deaths being 2568. The number of deaths in this new State from tuberculosis is over 9 per cent. of the entire number of deaths. Calculating the present population of the State at 800,000, we have a death-rate of thirty per hundred thousand.

From the outbreak of smallpox, which we had last year, there were seven deaths in the State. This outbreak cost the counties and cities of the State thousands of dollars, and people were willing to go to this necessary expense to stamp it out. Here we have a comparison of two hundred and forty-two deaths from tuberculosis, without any effort to stamp it out, or even to control or lessen its ravages; with seven deaths from smallpox, with stringent quarantine laws and plenty of funds available to eradicate it. Why is it that the people of this State, as well as of many others, allow this pennywise poundfoolish condition of affairs to continue? I believe it is because they are ignorant of the real existing conditions. They need to be educated regarding the danger and the control of tuberculosis, the same as they have been about smallpox. The very fact that the layman believes that tuberculosis is hereditary, instead of acquired, and that therefore nothing can be done to prevent the spread of the disease except through a law which prohibits the marriage of persons affected with tuberculosis, is the one great stumbling-block toward advancement. It is hard to make the people believe that tuberculosis is rarely hereditary. They think it incredible when the following statistics are quoted to them: That at the Munich abattoirs, from 1878 to 1882, the examinations of nearly one million of calves showed that only five were tuberculous, while during the same time the percentage in old cattle varied from 2 to 11 per cent.

It the way of control what should be done? I do not wish to say anything in regard to the control of tuberculous persons. That is a question which must be viewed from so many sides that there is not time to discuss it in this paper. But the control of cattle that supply food to man is a much simpler question. I have tried to show earlier in this article that tuberculous cattle played an important factor in the distribution of tuberculosis to man. As this is the case, and since Koch gave to the world a nearly certain test for the discovery of tuberculosis in cattle—the tuberculin-test—it seems that at least the milk-supply which forms such a large part of the food of the child might be protected for him by this method.

Regarding the supply of meat for food careful ante- and postmortem inspection will improve the value of meat as such. I need not go into details at this time how this should be done, as that will come later.

It is only a matter of time until the people will become educated in this and see the necessity of preventing the spread of this preventable disease. It is this latter burden of educating the people that will, like many previous ones have, fall on your shoulders as the family physician and adviser.

